# Lymph node metastasis around the entrance point to recurrent laryngeal nerve in papillary thyroid carcinoma

**DOI:** 10.1038/s41598-020-62031-w

**Published:** 2020-03-25

**Authors:** Tian Lv, Zhuoran Liu, Jiqi Yan

**Affiliations:** Department of General Surgery, Ruijin Hospital, Shanghai Jiao Tong University School of Medicine, Shanghai, 200025 China

**Keywords:** Thyroid gland, Thyroid cancer

## Abstract

There are few reports on the lymph nodes of entrance point to recurrent laryngeal nerve (LN-epRLN) in patients with papillary thyroid carcinoma (PTC). Thus, we investigated the clinical significance of LN-epRLN and implications it may have. An observational analysis of 878 consecutive patients with PTC who underwent thyroidectomy from April 2016 to March 2017 was conducted. We explored the surrounding tissue of laryngeal entry point, during routine central lymph node dissection (CLND). The lymph node specimens were sent separately for routine histopathological examination. Thereafter, complications and follow-ups were recorded. LN-epRLNs were found in 73 of the 878 patients, with the metastatic rate of 3.76%. Univariate and multivariate analysis showed central lymph node metastases can serve as independent predictors for LN-epRLN metastasis. In summary, we confirmed the significance of LN-epRLN in metastasis and recurrence, which required precise anatomy and thorough CLND. In PTC patients, especially in suspicious presence of central cervical lymph node metastasis, attention should be given to excising the nodal tissue at the laryngeal entry point.

## Introduction

Papillary thyroid carcinoma (PTC) is the most common type of endocrine malignancy yet^1^. Advocates of partial central lymph node dissection (CLND) cite the reduction in risks of postoperative complications^2^. Taken collectively, studies^3–5^ showed that the most common metastases and local recurrences of PTC nodal disease were in the central neck compartment. Additionally, recurrent malignancy after partial CLND may require a second surgery, which is considered more hazardous than a primary operation.

Reoperation on patients with regional recurrence at the laryngeal entry point would pose challenges due to its complexity^6^. Variations in the extent of adhesions around the lymph nodes of entrance point to recurrent laryngeal nerve (LN-epRLN)and neck muscle remnants make identification of the recurrent laryngeal nerve (RLN) and parathyroid glands more difficult compared to primary operation, and risks of certain postoperative complications, such as RLN palsy or hypoparathyroidism, are significantly higher^7^.

In this study, we evaluated a series of patients who underwent thyroidectomies with the aim of highlighting LN-epRLN metastasis in PTC patients.

## Methods

A total of 878 consecutive PTC patients received initial thyroidectomy at Shanghai Ruijin Hospital between April 2016 and March 2017. A prophylactic or therapeutic CLND was routinely performed in PTC patients. Exclusion criteria: those who underwent previous thyroid surgery; possess pathological types of thyroid carcinomas other than PTC; gross extrathyroidal extension invading the surrounding tissue of entrance point to RLN. According to the guidance of the 2018 Tumour Node Metastasis (TNM) staging system of American Joint Committee on Cancer/International Union Against Cancer (AJCC/UICC)^8^, we identified all the sample as T_1–3_N_0–1a_M_0_ PTC. All patients involved in this study gave their informed consent, and the data were anoymized. The study was approved by the medical ethics review committee in Shanghai Ruijin Hospital.

**Figure 1:**
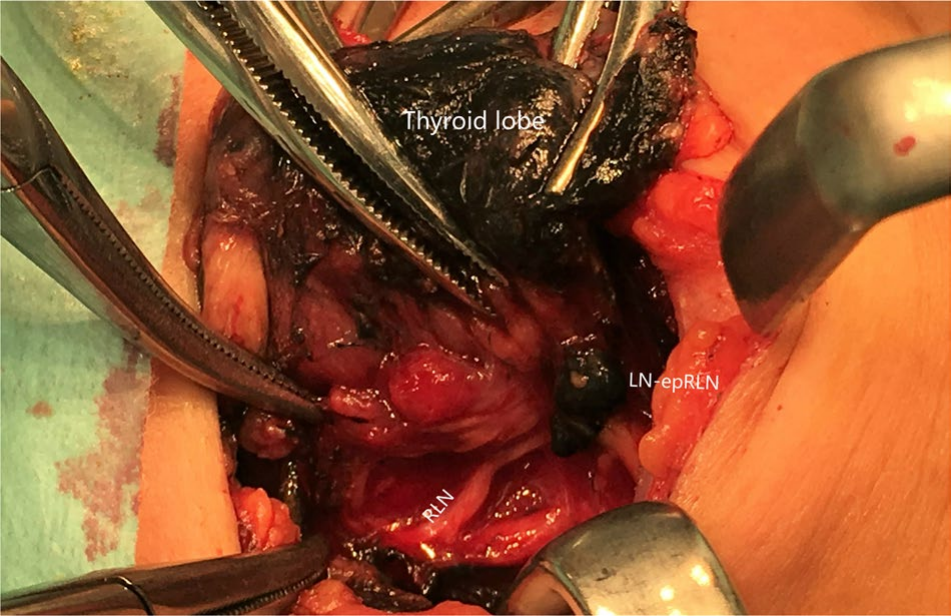
LN-epRLN area coverage. LN-epRLN, lymph node located within the distance from outer edge of the lymph node to RLN entrance point less than 5 mm.

All operations in our study were performed by the same group of surgeon (JQ Y.). Central lymph nodes included the Delphian, pretracheal, and paratracheal lymph nodes (LNs). We defined that LN-epRLN as lymph adipose tissue within 5 mm from the outer edge of the lymph node to the RLN entrance point. Steps for dissection of central LNs were as follows. Dissection of the lymph nodes around thyroid was completed at the same time as thyroidectomy. After thyroidectomy, the full length of RLN in the neck was revealed, and paratracheal lymph nodes (including LN-epRLN) were removed. All the surgical operations were performed in accordance with the relevant protocols and regulations. During the operation, Using a 1 ml syringe and a 27-gauge needle, approximately 0.1 ml of Carbon nanoparticles (Chongqing Lummy Pharmaceutical Co. Ltd., China) was slowly injected into the lobe. With gentle pressure applied, the surrounding lymph tissue, and the central-compartment LNs could be fully imaged in black^9^. The parathyroid glands were found to be visibly different from the thyroid glands and the LN tissues^10^ (Fig. 1).

All specimens of thyroid lesions were confirmed to be PTCs by postoperative pathological examination. The pre-operative and postoperative clinicopathological features were recorded, which included the following variables: age, gender, TgAb and TPOAb levels, primary tumor size and location, LN-epRLN laterality, thyroid nodules in primary lobe, and lymph node metastasis based on the final pathology report. 73 cases all had completed follow-up data (follow-up period from 24 to 36 months). The present study included the postoperative US examination and procedure-related complications.

### Statistical analysis

To identify differences between groups for specific variables, SPSS version 16 software (SPSS Inc, Chicago, IL) was used for statistical analysis. Univariate analysis was performed using Chi-square test. A *P*-value of <0.05 was considered to represent statistical significance.

## Results

In our experience, there were two metastatic lesions in the central compartment and the two patients suffered a second operation, which both resulted in the overlooked LN-epRLN. Our observational study of 878 consecutive patients with PTC was inspired by that.

LN-epRLNs were found in 73 of all the 878 patients (8.31%) and the metastasis rate was 3.76% (33/878). There were no significant difference in gender (*P* = 0.114) and age (*P* = 0.118) between the two groups (Table 1). For PTC patients presenting no LN-epRLN, 48.2% (388/805) had central LN metastatic rate, while 65.8% (48/73) PTC patients presenting LN-epRLNs had central LN metastases. Other central nodal metastases were more common in the positive LN-epRLN group (93.9 vs. 42.5%, *P* < 0.001). 38 patients were found to have elevated TgAb or TPOAb level, indicative of Hashimoto’s thyroiditis, among them, 15 patients (54.5%) had positive LN-epRLN. However, no significant relation was found between LN-epRLN metastasis and serum TgAb and TPOAb levels (P = 0.704) (Table 1).

**Table 1:** Univariate analysis showing clinical features associated with risk factors for LN-epRLN^a^ metastasis in the PTC patients^b^.

		LN-epRLN (+)	LN-epRLN (−)	P-value^d^
Total (patients)		33	40	
Age(years)		34.20 ± 10.34	40.00 ± 10.25	0.118
Gender	Male	9 (27.3%)	5 (12.5%)	0.114
	Female	24 (72.7%)	35 (87.5%)	
TgAb and TPOAb levels	Elevated	18 (54.5%)	20 (50.0%)	0.704
	Normal	15 (45.5%)	20 (50.0%)	
Other Lymph node metastases^c^	(+)	31 (93.9%)	17 (42.5%)	0.000
	(−)	2 (6.1%)	23 (57.5%)	

^a^LN-epRLN: lymph nodes of entrance point to recurrent laryngeal nerve.

^b^LN-epRLN was found in 73 of the 878 patients.

^c^Other Lymph node means the central lymph nodes, LN-epRLN excepted.

^d^P-value of <0.05 was considered to represent statistical significance.

LN-epRLN was identified bilaterally in 6 patients. Thus, there were 79 involved thyroid lobes in total. In this present study, we found that there were variant nodes of 1 to 4 (1.51 on average), with a positive rate of 46.84% (37/79). Then we analyzed the risk factors for LN-epRLN metastasis in the involved PTC thyroid lobes. The incidence of right LN-epRLN was higher than left (Right 41 vs Left 38). In univariate analysis, tumor located in the upper third (*P* = 0.044) and tumor multifocality (*P* = 0.033) were significant (Table 2). In our study, no significant relation to LN-epRLN metastasis was found with tumor size (*P* = 0.288).

**Table 2:** Univariate analysis showing clinical features associated with risk factors for LN-epRLN^a^ metastasis in the involved PTC thyroid lobes^b^.

		LN-epRLN(+)	LN-epRLN(−)	P-value^c^
Total (involved thyroid lobes)*		37	42	
Laterality	Left	16 (43.2%)	22 (52.4%)	0.424
	Right	21 (56.8%)	20 (47.6%)	
Multifocality^d^	(+)	25 (67.6%)	14 (33.3%)	0.033
	(−)	12 (32.4%)	28 (66.7%)	
Tumor Size	<10 mm	9 (24.3%)	17 (40.5%)	0.288
	10–20 mm	15 (40.5%)	15 (35.7%)	
	>20 mm	13 (35.1%)	10 (23.8%)	
Tumor Location	upper 1/3	25 (67.6%)	15 (35.7%)	0.044
	lower 2/3	12 (32.4%)	27 (64.3%)	

^a^LN-epRLN: lymph nodes of entrance point to recurrent laryngeal nerve.

^b^LN-epRLN was identified bilaterally in 6 of the73 patients. Thus, there was 79 involved thyroid lobes in total.

^c^P-value of <0.05 was considered to represent statistical significance.

^d^Defined as more than one PTC in the same lobe as the primary carcinoma by the postoperative pathology.

**Figure 2:**
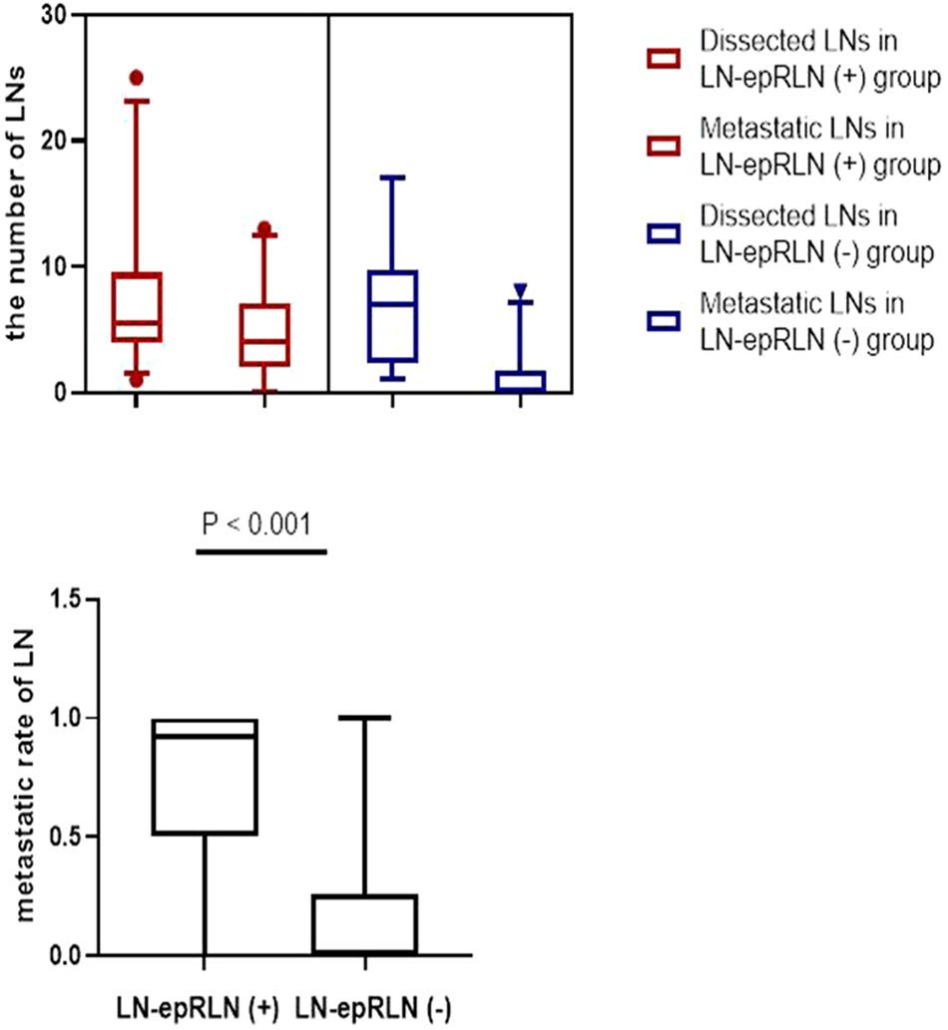
To analyze disease burden of PTC patients, the number of dissected and metastatic LNs were recorded. In our study, central LN metastasis was stable parameters pointing toward positive LN-epRLN.

Further, multivariate analysis (Table 3) displayed that tumor location (OR = 1.957, 95% CI: 0.797–4.784, *P* < 0.05) and other central nodal metastases (OR = 20.971, 95% CI: 4.401–99.924, *P* < 0.001) may be independent risk factors. In line with multivariate analysis, central LN metastasis was stable parameters pointing toward positive LN-epRLN (Fig. 2).

**Table 3:** Multivariate analysis showing clinical features associated with risk factors for LN-epRLN^a^ metastasis in the involved PTC thyroid lobes^b^.

	OR (95% CI)	P-value^c^
Laterality (Left vs Right)	0.511 (0.209–1.254)	0.178
Multifocality	1.750 (0.717–4.272)	0.057
Tumor Size (<20 mm vs > 20 mm)	1.733 (0.651–4.617)	0.325
Tumor Location (upper 1/3 vs lower 2/3)	1.957 (0.797–4.784)	0.046
Other Lymph node metastases^e^	20.971 (4.401–99.924)	0.000

^a^LN-epRLN: lymph nodes of entrance point to recurrent laryngeal nerve.

^b^LN-epRLN was identified bilaterally in 6 of the73 patients. Thus, there was 79 involved thyroid lobes in total.

^c^P-value of <0.05 was considered to represent statistical significance.

^d^Defined as more than one PTC in the same lobe as the primary carcinoma by the postoperative pathology.

^e^Other Lymph node means the central lymph nodes, LN-epRLN excepted.

No patient had permanent RLN injury or permanent hypoparathyroidism in this study, and no patient had a second operation during the follow-up period. Seven (0.8%) patients had transient hoarseness (4–6 weeks), which were checked under fiberlaryngoscopy at one month and 6 months after surgery. Six (0.7%) patients had postoperative transient hypoparathyroidism (5–14 days).

## Discussion

Previous literatures^3–5^ have shown that LN involvement is relevant to local recurrence. Because of the increased risk of recurrence with focal “berry-pickin” techniques, compartmental surgery is recommended in accordance to the current American Thyroid Association (ATA) guidelines^11,12^. Our study was inspired by two cases where the PTC patients suffered hoarseness after a second operation addressin the presence of metastatic LN-epRLN, which may have been reserved in consideration of RLN injury and hypoparathyroidism during the primary operation.

The anatomic boundaries of CLND are from the hyoid bone to the innominate artery. An appropriate range of CLND should both reduce the local recurrence rate and the incidence of post-operative complications^11^. In CLND, fear of RLN injury and hypoparathyroidism may lead to insufficient exposure where metastases are left at the laryngeal entry point. We refer to these as involved LN-epRLNs in our study. To this date, no studies have covered the significance of LN-epRLN, as part of central LNs^13,14^.

LN-epRLN was defined as lymph adipose tissue within 5 mm from the outer edge of the lymph node to the RLN entrance point (Fig. 1). In our study, there were variant nodes, 1 to 4 (1.51 on average), in diameter between 6 mm and 10 mm. In the present study, LN-epRLN was found in 73 of 878 patients (8.31%) and the metastatic rate was 3.76% (33/878). After the Carbon nanoparticles mapping, the non–black-stained parathyroid glands were easily discriminated from the lymph-fatty tissue^9,10^. Our findings indicated that CLND could be performed safely with the use of intraoperative CN mapping, Which has been reported in the previous study^10^.

In terms of involved thyroid lobes, the positive LN-epRLN rate was 46.84% (37/79), and the incidence of the right side was slightly higher than that on the left side. The left and right RLNs have slight differences in anatomical position, and the cervical part of the esophagus located closely next to the left. Therefore, there was a triangular space located posterior to the right RLN, which makes space for lymph adipose tissue^15–17^. In the literatures, the presence of Hashimoto’s thyroiditis leads to reactive lymphaden proliferation^18,19^. We preferred that the patients of Hashimoto’s thyroiditis would have positive LN-epRLNs. However, it showed no statistical significance between LN-epRLN metastasis and serum TgAb/TPOAb levels in our study, limited by small samples.

The univariate analysis showed that the factors influencing LN-epRLN involvement were the other central LN metastases, the upper third tumor location and tumor multifocality. Previous studies^20–24^ have found that ipsilateral multifocal disease could be used to predict neck lymph node metastases, which may reflect the ability of clonal formation of cancer cells^25–28^. This result strengthens the argument for CLND (LN-epRLN included) in PTC as preoperative US and intra-operative frozen biopsy shows multifocality of the PTC nodules. In our study, the rate of other central LN metastasis in the presence of LN-epRLN metastasis was 93.9%. In line with multivariate analysis, central LN metastasis was stable parameters pointing toward positive LN-epRLN (Fig. 2), which can be evaluated through preoperative US and frozen biopsy performed intra-operatively.

Usually, most patients with PTC obtain a 10-year survival rate of 80–90%, but the regional recurrence rate after surgery is 5–20%^12^. Therefore, it’s important to improve the thoroughness of CLND. Our results mirror the data from previous literatures: reoperative CLND has been shown to have rates of RLN injury 21% transiently and postoperative hypocalcemia with a large range of 0–24%^29–36^. However, the incidence of complications varies according to the skill and experience of the surgeon^7,37^.

PTC patients with clinically involved LNs in the central compartment should be managed with a LN-epRLN dissection during CLND, taking into consideration RLN and parathyroid glands which are closely related to the nodal basins. The standard exploration and resection procedures are key factors that impact the relative difficulty of performing secondary surgery.

Our observational study was limited by small samples, but we focused on acknowledgement of LN-epRLN. Moreover, patients with lateral neck lymph node matastases were excluded due to inclusion criteria. These patients may have experienced worse outcomes than patients enrolled in our study. However, to our knowledge, this is the first article in English to progress our understanding of LN-epRLN metastases in PTC patients.

## Conclusions

In summary, we confirmed the significance of LN-epRLN in metastasis and recurrence, which required precise anatomy and thorough CLND. In PTC patients, especially in suspicious presence of central cervical lymph node metastasis, attention should be given to excising the nodal tissue at the laryngeal entry point.
